# Suspected Pediatric-Onset Common Variable Immune Deficiency (CVID) in a Seven-Year-Old Female With Pulmonary Manifestations

**DOI:** 10.7759/cureus.29703

**Published:** 2022-09-28

**Authors:** Noshaba Noor, Mariam Ghori, Rameen A Molani, Mohsina N Ibrahim

**Affiliations:** 1 Department of Paediatrics, National Institute of Child Health, Karachi, PAK; 2 Department of Medicine, Jinnah Sindh Medical University, Karachi, PAK; 3 Department of Paediatrics and Endocrinology, National Institute of Child Health, Karachi, PAK

**Keywords:** common variable immunodeficiency, intravenous immunoglobulins (ivig), hematopoetic stem cell, auto immune, lymphocytic count, recurrent infections, hypogammaglobulinemia, genetic panel testing, sinopulmonary

## Abstract

Common variable immune deficiency (CVID) is the most common of all primary immunodeficiency rare diseases characterized by hypogammaglobulinemia. This is caused by the defective functioning of B-cells and T-cells, resulting in recurrent infections. Its etiology is unknown but most commonly initiated due to epigenetic factors and epistatic interactions. Moreover, it has a bimodal age distribution and can be more evident from infancy to after 4th decade of life.

Herein, a seven-year-old female, the first product of consanguineous marriage with no family history of immunodeficiency disorders, presented predominantly with sinopulmonary involvement. It manifested as severe pulmonary pneumonia, atelectasis, patchy alveolar infiltrates, and lung nodules. She also had a history of diarrhea and otitis media. Despite having a history of recurrent infections since three years of age, she was diagnosed late due to a lack of awareness and knowledge about the presentation of CVID and its different manifestations among the medical community in Pakistan. The diagnosis of CVID is based on the clinical and immunological manifestation of the patient with respect to the European Society of Immune Deficiencies (ESID) diagnostic criteria. Therefore, genetics help detect mutations leading to CVID and establish a genetic diagnosis for CVID-like disorders. However, genetic panel testing is not used as a diagnostic tool in Pakistan due to the unavailability of resources. Instead, the clinical presentation, abnormal lymphocytic counts, and immunoglobulin levels may help diagnose CVID.

Early diagnosis will help in the timely utilization of the most effective treatment and management options available. These include intravenous immunoglobulin (IVIG) and hematopoietic stem cell therapy. Ig replacement therapy has shown a beneficial role in halting the cycle of recurrent infections and improving the prognosis of CVID. However, it's a bit expensive therapy. Moreover, the role of hematopoietic stem cell therapy in treating CVID has been documented, but it's not so common and practical.

## Introduction

Common variable immune deficiency (CVID) is a heterogeneous disorder presenting various clinical manifestations such as autoimmune cytopenias, lymphoproliferation, and granulomas [1]. In addition, it may involve various genetic abnormalities in 10-15% of the cases [2]. Still, its pathogenic involvement has not been established yet, with most commonly involving TNFRSF13B encoding for transmembrane activator calcium modulator and cyclophilin ligand interactor (TACI), inducible T cell costimulator (ICOS), B-cell activating factor receptor (BAFF-R), and CD-19 [2]. Moreover, it is characterized by abnormal immunoglobulin production, mainly immunoglobulin G (IgG) and occasionally IgA and IgM, due to defective B-cell differentiation leading to recurrent infections [3,4].

It has a prevalence of 0.001 to 3.374 per 100,000, making it a rare yet the most common primary immunodeficiency in humans [5]. While it can occur at any age, it is usually classified into the pediatric age group, which is <18 years of age, and the adult age group >18 years of age [6].

CVID can have infectious and non-infectious manifestations (autoimmune diseases, malignancy/ inflammatory diseases such as benign lymphocytic infiltration or granulomatous diseases, and sinopulmonary, gastrointestinal, and central nervous system involvement) [7].

Diagnosis for CVID is challenging in developing countries due to the lack of sensitivity and specificity of the available diagnostic criteria and the lack of physician knowledge. Although CVID is a clinical diagnosis, specific testing is necessary to rule out monogenic CVID-like disorders [3].

The treatment may differ in patients due to the involvement of various organ systems depending upon the clinical presentation and manifestation of symptoms [3]. However, the most effective management modalities include immunoglobulin replacement therapy and immunosuppressive therapy. At the same time, autologous hematopoietic stem cell transplantation (HSCT) has been documented to show significant results as a treatment modality, but it's not so common [8].

## Case presentation

A seven-year-old Pakistani female, a resident of Karachi, Pakistan, presented to the National Institute of Child Health (NICH) with complaints of fever and cough for the last two weeks. According to her mother, the patient developed high-grade undocumented, intermittent fever not relieved with antipyretics. She also had a productive cough with mucoid sputum, for which she took oral amoxicillin-clavulanic acid; however, no improvement was noticed. A day before arriving at the hospital, she had an episode of hemoptysis, fresh blood mixed with sputum. They visited a local doctor who referred them to NICH. Her past medical history was significant for on and off flu-like symptoms, two episodes of ear discharge in the last two years, and two to three episodes of large volume watery diarrhea each year since three years of age, for which she had been treated on oral medications. There is also a history of hospital admission due to pneumonia at three years of age in a private hospital where she required oxygen support and IV antibiotics. At five years of age, she again had similar complaints and was offered hospital admission, but her parents were reluctant to admission; hence she was treated with a daily course of IV antibiotics at a local clinic.

According to her mother, she has always been thin and lean and has not gained adequate weight in the last few years as her other siblings did. She had no known allergies and was developmentally appropriate for her age. Her birth history was unremarkable, and she had been vaccinated to date according to Pakistan’s vaccination’s schedule. She is the first twin product of consanguineous marriage, and her other siblings were both healthy and alive. There is no history of any severe illness in the family.

Her physical examination revealed 92 beats/min pulse, blood pressure 100/70 mmHg, temperature 99°F, and 40 breaths/min respiratory rate. She had grade 2 clubbing on General Physical Examination, and her anthropometric measurements revealed that her height (110 cm) and weight (17 kg) were lying at less than the third centile. On respiratory examination, she had asymmetrical chest movements with decreased chest expansion on the left side and dull percussion notes over the left lower chest from the fourth to seventh intercostal space. Also, she had bronchial breath sounds, crackles, and increased vocal resonance in the left mid and lower zones. The rest of the systemic examination was unremarkable.

Her laboratory data revealed an elevated WBC count of 22x10^9^/L (Normal range: 4.5-13.5x10^3^) with neutrophilia of 64% and an absolute neutrophilic count of 14080. Her blood and sputum culture were negative; however, C-reactive protein (CRP) and erythrocyte sedimentation rate (ESR) was elevated at 6.8 mg/L (Normal range: 3-5 mg/L) and 95 mm/hr (Normal range: <15 mm/hr). Her serum blood urea nitrogen, creatinine, electrolytes, and liver function tests were within normal limits.

**Figure 1:**
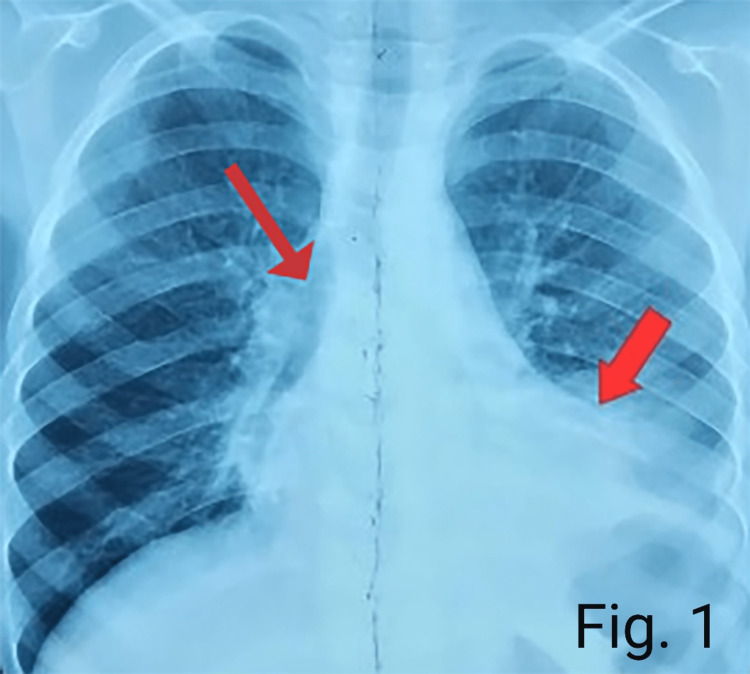
Chest X-ray suggesting air bronchogram, silhouette sign and patchy alveolar infiltrates in both the lung fields.

**Figure 2:**
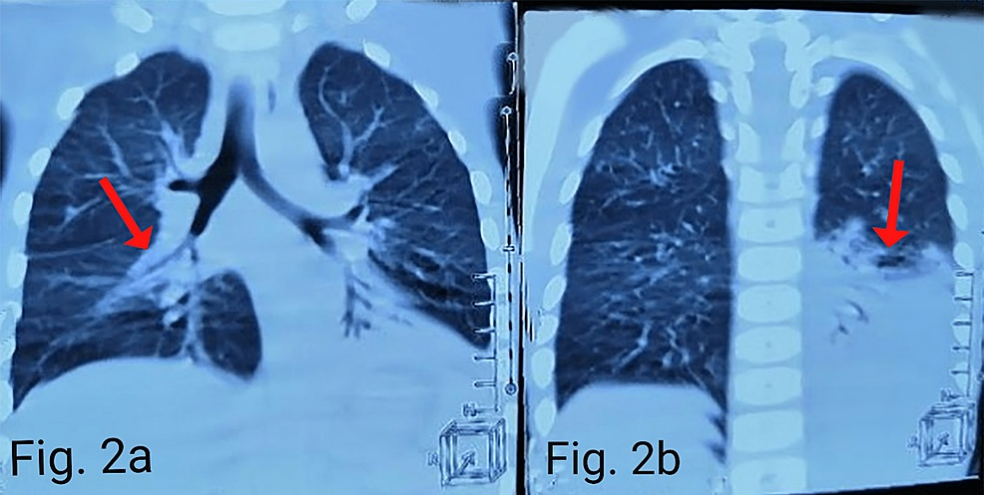
CT scan (coronal view) of the chest showing large areas of consolidation/atelectasis in the left lower lobe (a) and small areas of consolidation in the right middle lobe (b).

**Figure 3:**
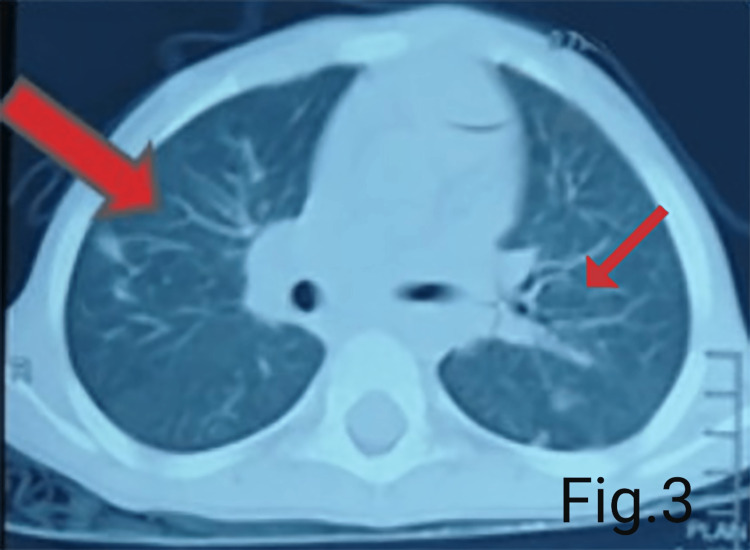
A transverse cut in CT scan of the chest showing few nodular areas in the right upper, middle and lower lobes and left lingular segment.

Chest X-ray suggested patchy alveolar infiltrates scattered in both the lung fields more on the left side of the chest. An air bronchogram and a silhouette sign were present (Figure 1). CT scan chest (Figure 2) showed large areas of consolidation/atelectasis in the left lower lobe and small areas of consolidation in the right middle lobe, left lingular segment, and both lower lobes. Few nodular areas were seen in the right upper, middle and lower lobes and left lingular segment (Figure 3). Findings were suggestive of pulmonary infection.

Her ΔF508 mutations were sent, but it was not detected, and pancreatic fecal elastase levels were 283 ug/ml (Normal range: >200 ug/ml). Human immunodeficiency serology was negative. Serum immunoglobulin assay showed a decrease in IgA and IgG levels as <0.15 g/L (Normal range: 0.33-2.02 g/L) and 1.29 g/L (Normal range: 6.33-12.8 g/L), respectively; however, IgM levels were within the normal range (0.48-2.07 g/L). It raised the suspicion of an immunodeficiency disorder, and a lymphocyte subset analysis was ordered. The analysis revealed low absolute counts of CD3+, CD8+ T-lymphocytes, CD19+ Total B- lymphocytes, and CD56+ Natural Killer cells. CD4+ T-helper lymphocytes were within normal limits, and the CD4+/CD8+ ratio was increased.

**Figure 4:**
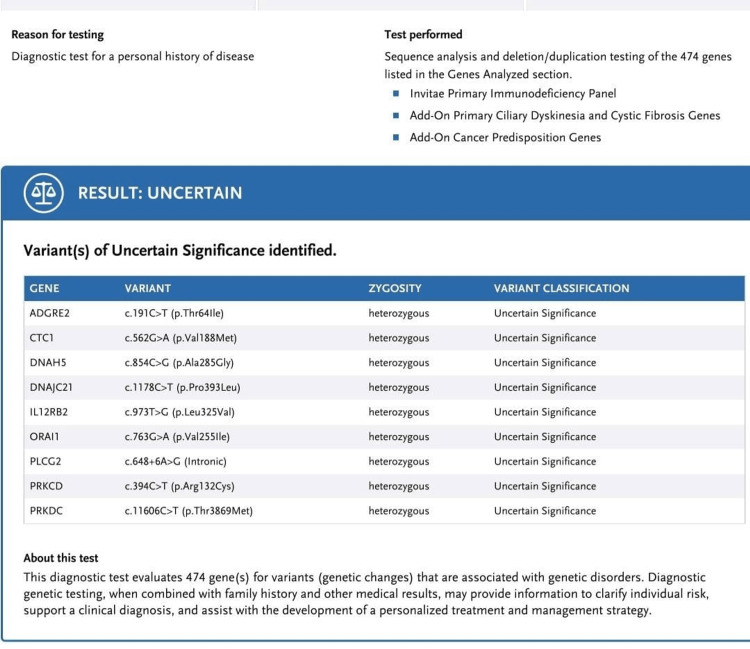
Genetic panel testing showing variant of uncertain significance presenting heterozygosity for the genes linked with CVID, including CTC1, PRKDC, PRKCD, PLCG2, and DNACJ21. CVID: Common variable immune deficiency

After discussing the case with an infectious disease specialist, we treated our patient with a tazobactam/piperacillin combination. Parents were counseled regarding the need for genetic testing and the possibility of a bone marrow transplant. After discussing with her parents, a genetic panel was ordered. It showed variants of uncertain significance (VUS) presenting heterozygous mutations in genes linked with CVID, including CTC1, PRKDC, PRKCD, PLCG2, and DNACJ21 (Figure 4). These genes were found in patients with CVID, but their definitive role in CVID is not established. We strongly suspected common variable immunodeficiency based on the history of recurrent sinopulmonary infections, hypogammaglobulinemia, lymphocyte subset analysis, and genetic panel test. An allergist-immunologist was taken on board, and intravenous immunoglobulin was given. After a discussion with a geneticist, her parent’s genetic samples were also ordered. These panels revealed paternal heterozygosity in genes DNAH5, DNAJC21, IL12RB2, PRKCD, and PRKDC. Moreover, maternal heterozygosity was seen in ADGRE2, CTC1, and DNAJC21. Follow-up visits were advised to ensure a monthly maintenance dose of intravenous immunoglobulin, but the patient lost to follow-up.

## Discussion

While CVID is the most common primary immunodeficiency in Pakistan, it is still not diagnosed and reported on time due to overlapping symptoms with other clinical conditions. Due to this, doctors are even unable to classify it as a differential. CVID is characterized by hypogammaglobulinemia due to inadequate functioning of B-cells and T-cells, which subsequently causes recurrent infections, especially sinopulmonary infections [3]. The etiology of most CVID cases is unknown; however, 5%-25% of its cases are found particularly in consanguineous populations [3].

While CVID can occur in any age group, it is usually classified as pediatric and adult-onset [6]. The clinical manifestation of infectious diseases in CVID remains uniform irrespective of age and most commonly includes pneumonia, sinusitis, otitis media, bronchitis, and shingles [6]. Our patient displayed similar symptoms and had a history of sinopulmonary involvement since birth, otitis media, diarrhea, and pneumonia in the last few years. She was given symptomatic treatment as the symptoms manifested individually over the years. She was hospitalized once due to severe pneumonia at the age of three. Though she presented with recurrent infections throughout, the doctors could not even suspect it as CVID due to a lack of awareness among the physicians and its rare nature in the Pakistani population.

It has been reported that the leading cause of mortality in CVID patients is due to pulmonary complications [1]. The pulmonary complications present as interstitial pneumonia, bronchiectasis, nodules, and interstitial lung disease in pediatric-onset CVID [6]. In the pediatric age group, bronchiectasis secondary to CVID is most commonly known to cause male death. In contrast, lung nodules and pneumonia secondary to CVID are primarily associated with female deaths [6]. Similarly, our female patient presented with few nodular areas in the lung and severe pneumonia. In addition, she also showed patchy alveolar infiltrate in both the lung fields in the X-ray chest. In contrast, in the chest CT scan, atelectasis was seen in the left lower lobe and small areas on consolidations in the right middle and lower lobes. All this suggested pulmonary infection.

Abnormalities of the lymphocytic subset in CVID patients have been reported, such as low switched memory B-cells and expansion of CD21, low CD4+ T cells, B cells, Natural killer cells, and CD8+ T cells [1]. Similarly, our patient also showed low absolute counts of CD19+ Total B- lymphocytes, CD3+, CD8+ T-lymphocytes, and CD56+ Natural Killer cells. However, her CD4+ cells were within the normal range. Unfortunately, due to affordability issues of the patient, her complete peripheral lymphocytic subset was not done. Moreover, according to European Society of Immune Deficiencies (ESID) criteria, hypogammaglobulinemia is a definitive diagnosis in CVID patients with low IgA and IgG levels, while low IgM in few patients and low switched memory B-cells or absent isohemagglutinins, or poor vaccine responses, and clinical presentation of CVID [3]. Similarly, our patient presented with low IgA and IgG levels, but contrary to the usual presentation, IgM levels were within the normal range. Therefore, the abnormal lymphocytic subset, hypogammaglobulinemia, and clinical presentation suggest CVID in our patient.

Later, the genetic panel test was ordered, showing a heterozygous variant of uncertain significance for the genes ADGRE2, CTC1, DNAH5, DNAJC21, IL12RB2, ORAI1, PLCG2, PRKCD, and PRKDC. The parents' genetic panel clearly showed our patient's polygenic origin of CVID. This suggested the involvement of a more complex inheritance pattern than the monogenic one [9]. Therefore, these genetic defects may vary according to the clinical manifestation of the patient [9]. Furthermore, since the parents are in a consanguineous marriage, the likelihood of gene mutations in their offspring increases, as seen in the patient [10]. CVID is often misdiagnosed due to no proper criteria for its diagnosis and poor knowledge among the doctors in Pakistan. However, in other parts of the world, ESID diagnostic criteria are used to diagnose CVID. Moreover, the clinical picture of CVID is similar to other immune disorders making the diagnosis even more difficult [3]. Therefore, whenever patients present with recurrent infections with abnormal lymphocytic subsets, the doctors should always think of CVID as a differential in such a case. In addition, a genetic panel is not a diagnostic tool for CVID, but it can direct toward the monogenic CVID-like disorder. Unfortunately, no genetic panel testing is available in Pakistan, and the lymphocytic subset analysis is quite expensive. The rural areas are even deprived of basic diagnostic tests such as the lymphocytic subset analysis, due to which they are oblivious to this disease. So, recurrent infections and immunological features in any pediatric patient should be one of the main indications for the diagnosis of CVID.

Our case is similar to cases reported in the past where the diagnosis of CVID was established on clinical grounds. An example is a case report from Pakistan published in 2011 where a young Asian male with recurrent pneumonia was diagnosed with common variable immunodeficiency in adulthood on clinical grounds and basic investigations [11]. Another case of an 11-year-old female was reported with a history of recurrent infections in childhood. Based on clinical grounds, she was managed as a case of common variable immunodeficiency [12].

The management of CVID mainly depends upon the symptoms and the systemic involvement in the patient [3]. However, the primary treatment options include high-dose intravenous immunoglobulin (IVIg), steroids for CVID cytopenia, and anti-D antibodies [3]. However, for the vast majority of CVID patients, particularly with infectious manifestations, hematopoietic stem cell transplantation can be used to treat severe auto-immune manifestations of CVID [8]. Our patient was treated with intravenous immunoglobulin and was advised for genetic testing and a bone marrow transplant. Early diagnosis of CVID is essential in treating and preventing the progression of CVID. Early treatment with IVIg has a beneficial role in infectious complications, but its contribution to immune dysregulation, such as auto-immune manifestations, is ambiguous. Also, the doctors should go for prophylactic treatment when the patients present with severe recurrent infections, abnormal lymphocytic subset, and hypogammaglobulinemia.

## Conclusions

CVID is a rare primary immunodeficiency that involves multiple organ systems, most importantly sinopulmonary. It presents recurrent infections, abnormal low switch memory B-cells and expansive CD21 cells, and hypogammaglobulinemia. In addition, it may have an association with epigenetic factors. Therefore, in countries like Pakistan, where genetic testing is unavailable, the clinical presentation of symptoms and tests to evaluate lymphocytic counts and immunoglobulin levels can assist in diagnosing CVID among many differentials. This will lead to the utilization of the most effective treatment, hematopoietic stem cell therapy, to increase the life expectancy of the patients. Moreover, there is a further need for research on the complex form of the inheritance pattern and the role of environmental factors in CVID.
